# Developing an instrument to measure effective factors on Clinical Learning

**Published:** 2016-07

**Authors:** IDEH DADGARAN, MANDANA SHIRAZI, AEEN MOHAMMADI, ALI RAVARI

**Affiliations:** 1 Medical Education Research Center (MERC), Guilan University of Medical Sciences, Rasht, Iran;; 2 Department of E-learning in Medical Education, Virtual School, Center of Excellence for E-learning in Medical Education, Tehran University of Medical Sciences (TUMS), Tehran, Iran;; 3 Education Development Center, Medical Education Department, Tehran University of Medical Sciences, Tehran, Iran;; 4 Department of Clinical Science and Education, Södersjukhuset, Karolinska Institutet, Stockholm, Sweden;; 5 Geriatric Care Research Center, Rafsanjan University of Medical Sciences, Rafsanjan, Iran

**Keywords:** Learning, Clinical, Teaching, Nursing student

## Abstract

**Introduction:**

Although nursing students spend a large part of their learning period in the clinical environment, clinical learning has not been perceived by its nature yet. To develop an instrument to measure effective factors on clinical learning in nursing students.

**Methods:**

This is a mixed methods study performed in 2 steps. First, the researchers defined “clinical learning” in nursing students through qualitative content analysis and designed items of the questionnaire based on semi-structured individual interviews with nursing students. Then, as the second step, psychometric properties of the questionnaire were evaluated using the face validity, content validity, construct validity, and internal consistency evaluated on 227 students from fourth or higher semesters. All the interviews were recorded and transcribed, and then, they were analyzed using Max Qualitative Data Analysis and all of qualitative data were analyzed using SPSS 14.

**Results:**

To do the study, we constructed the preliminary questionnaire containing 102 expressions. After determination of face and content validities by qualitative and quantitative approaches, the expressions of the questionnaire were reduced to 45. To determine the construct validity, exploratory factor analysis was applied. The results indicated that the maximum variance percentage (40.55%) was defined by the first 3 factors while the rest of the total variance percentage (59.45%) was determined by the other 42 factors. Results of exploratory factor analysis of this questionnaire indicated the presence of 3 instructor-staff, students, and educational related factors. Finally, 41 expressions were kept in 3 factor groups. The α-Cronbach coefficient (0.93) confirmed the high internal consistency of the questionnaire.

**Conclusion:**

Results indicated that the prepared questionnaire was an efficient instrument in the study of the effective factors on clinical learning as viewed by nursing students since it involves 41 expressions and properties such as instrument design based on perception and experiences of the nursing students about effective factors on clinical learning, definition of facilitator and preventive factors of the clinical learning, simple scoring, suitable validity and reliability, and applicability in different occasions.

## Introduction

The learning methods of the nursing students are among the most important concerns of the educational authorities. Clinical learning is among the important issues resulting in perception of the importance of nursing students’ performance in the clinical environment and its effect on expansion of nursing career. Within the clinical learning, the student bridges between his/her new and old experiences as a cyclic process (1). “Nursing experience in clinical environment is clinical learning” states Ogier (1989) (2). Learning is an active process in which the instructor plays a facilitating role. So, detection of the effective factors on learning process is very important and enables the instructor to offer a more efficient teaching (3). Clinical learning is affected by a large number of factors including individual attitude, experience, and characteristics, psychomotor skills, problem-solving ability and knowledge of the student, physical structure of the environment, educational content, and method (4-6). In a study conducted on perception of nursing students of clinical learning, 5 effective factors were detected: student-staff relationship, commitment of the nursing managers towards teaching, patients’ communication, students’ satisfaction, hierarchical structure, and religious factors (4). The real learning of the nursing students happens in the clinical environment and the students learn how to manage their time and readiness for clinical experiences and how to complete professional completion steps from dependence to independence. The quality of the achieved experiences is highly affected by the clinical situations introduced to the students (7, 8).

The diversity of the clinical environments, experience in the real environment, gap between theory and practice, and need for improving the clinical nursing education highlights the necessity to perform studies in this area. Considering the mentioned points, the researchers of this work made an attempt to organize a mixed qualitative-quantitative study using the interviews and content analysis on effective factors on clinical learning in students’ perspective. Here, qualitative content analysis can be employed to evaluate the experiences of the students and extract their opinion on clinical learning and then prepare a questionnaire for further studies through defining concepts such as the students’ perspective on clinical learning and further detection of the background and factors associated with clinical learning. 

## Methods

This is a mixed method study performed to develop an instrument to measure effective factors on clinical learning in nursing students. Mixed methods is a rich field for the combination of data because with this design words, pictures, and narrative can be used to add meaning to the numbers. In other words, what we generally consider qualitative data words, pictures, and narrative can be combined with quantitative, numerical data from a larger-scale study on the same issue, allowing our research results to be generalized for future studies and examinations (9).

In the first step, nursing students perceptions about clinical learning and its affecting factors were researched using the qualitative content analysis approach and the main themes and items of the questionnaire were extracted from the data obtained from the interviews. In this step, and based on the study objectives, the participants were selected among the nursing students in the fourth semester on. Sample selection process continued until data saturation (choosing 22 students). The data were gathered using the semi-structured individual interviews performed by the main researcher and analyzed using the qualitative content analysis

In the second step, using the concepts extracted from first step, the main themes, items, and expression of the questionnaire were defined. For face validity of the work, two qualitative and quantitative approaches were employed. For qualitative face validity, face-to-face interviews were performed with 10 students and factors including difficulty level (difficulty of understanding the expressions and words), proportionality level (the desired proportion and relationship between the items and aspects of the questionnaire), and ambiguity (likelihood of misunderstanding the items or presence of ambiguity in the words) were investigated. After correcting the items based on the students’ opinion, the quantitative item impact method was applied to reduce the improper expressions and determine the importance of each expression (10). For each item, five-point Likert scale was used as: “completely important” (5 scores), “rather important” (4 scores), “medium important” (3 scores), “little important” (2 scores), and “not important at all” (1 score). Through the item impact method, once the effect score is greater than or equal to 1.5, the expression is recognized as appropriate for the further analyses and is kept (10, 11). The expressions were scrutinized for several times by the research team members and the suggestions were employed and then reviewed by two experts in Farsi literature. Also, to determine the content validity,we used both qualitative and quantitative approaches. Content validity was determined through the judgment of the experts and students. For qualitative content analysis, the researcher asked 10 experts to give their feedback after qualitative evaluation of the questionnaire. For quantitative content validity analysis, content validity ratio (CVR) and content validity index (CVI) were used. To determine CVR, 15 experts (different from the previously mentioned ones) were asked to evaluate each item based on 3-part scale (composed of expressions including “it is necessary”, “it is useful but not necessary”, and “it is not necessary”). According to the Lawshe tables (1975), the minimum value of CVR was determined 0.49. Then, CRI was determined based on the approach proposed by Walts & Bassel (1983). In this regard, the researcher offered the constructed questionnaire to the experts to assess the relevance, simplicity, and clarity of the expressions in the questionnaire. So, these three criteria (relevance, simplicity, and clarity) were examined by 15 experts (again different ones) in a 4-point Likert scale. CVI score obtained in this work for each item was calculated by dividing the number of experts agreeing with the expression with scores 3 and 4 by the total number of experts. Hyrkäs et al. recommended a score of 0.79 and higher for items reception based on CVI score (12).

Next, the mean CVI of the questionnaire (S-CVI/Ave) was calculated based on the mean CVI scores of all the expressions. In addition, Polit et al. recommended a score of 0.9 and higher for S-CVI/Ave acceptance (13). Finally, the expressions were organized in the form of three themes including human resources of clinical learning (29 expressions), learning conditions (4 expressions), and clinical learning strategies (12 expressions). The questionnaire was designed based on 5-point Likert scale (“completely agreed”, “agreed”, “I have no idea”, “disagreed”, and “completely disagreed”). In this method, a score of 1-5 was assigned for each response. As the final analysis, the items “completely agreed” and “agreed” as well as “completely disagreed” and “disagreed” were integrated.

After applying the required changes and designing the questionnaire with 5-point Likert scale, exploratory factor analysis (EFA) – which evaluates the internal relationship of the parameters – was applied to determine the construct validity of the items obtained from the interviews in order to detect the categories from the parameters with the highest relationship. In this regard, factor analysis is considered among the very important steps in designing the novel tools (14). The number of required samples for performing factor analysis for defining the construct validity is different in the view of different researchers. The number of recommended samples for factor analysis is 5-10 for each expression of the questionnaire. However, some experts believe even 3 samples for each expression suffice in the case the mentioned variance percentage and loading is greater than 0.8 (15). Factor analysis is a technique to determine a category of related questions in a given scale. Each category or factor includes a group of parameters which have higher correlation between themselves compared to the parameters rather than them alone. Each factor explains a rather similar property and can be interpreted through the categorization of parameters (10). In the present study, EFA was applied using the Kaiser Meyer Olkin (KMO) sampling index and Cruet-Bartlett’s tests and principle component analysis (PCA) on 45 expressions. Here, the number of elected samples were 5 times to the items of expressions (227 students). The value obtained from KMO test varied in the range of 0-1, where the higher values imply better factor analysis. Here, the values greater than 0.80 are considered as suitable (14). In the next step, the factors were extracted after calculation of the covariance matrix between the parameters. The factors latent in the instrument were extracted through the PCA technique. There are various rules for determination of the number of factors in PCA. To this aim, in this research scree plot and Eigen value techniques were used. In this study, categorization criterion of the factors was considered based on the cutoff point of 0.35, as the minimum required value for keeping the expression in the parameters extracted from factor analysis. Besides, the Eigen value was considered as greater than 2. Furthermore, varimax which is one of the orthogonal rotations was applied for simplification and interpretability of the factor components (14).

Finally, after validation of items a questionnaire with 41 expressions was constructed based on the nursing students’ perspective about effective factors on clinical learning. After designing the questionnaire, it was distributed among 20 nursing students in the fourth semester and higher to determine its validity. The reliability of the questionnaire was defined using the α-Cronbach coefficient. 

## Results

Qualitative content analysis of the data obtained from interviews with nursing students led to 1198 first-level codes extracted from the analysis of the interviews; three main themes with 9 main categories were specified: Human resources of clinical learning, Clinical learning conditions and Strategies of clinical learning (Table 1).

**Table 1:** Themes and their main categories emerging from analysis of nursing student’s views on effective factors in clinical learning

**Themes**	**Main categories**
**Human resources of clinical learning**	Community and culture
	Student
	Teacher
	Staff
	Colleagues
**Clinical learning conditions**	Physical factors
	Management factors
**Strategies of clinical learning**	Clinical skills improvement
	Theoretical skills improvement

Based on the defined concepts and literature review of the available references on clinical learning of the nursing students, a group of desired items was obtained based the themes emerging from the interviews. After performing the interviews, a total number of 102 items were extracted from the qualitative phase. Then, the extracted items were examined in three meetings and the expressions with overlapping concepts were merged. After this step, the number of expressions in the questionnaire was reduced to 81. To determine the qualitative face validity of the items, the required modifications were applied based on the students’ opinion. Then, in the next step irrelevant expressions were eliminated to reduce the number of expressions. After that, the quantitative item impact method was employed to determine the importance of each expression, where 20 expressions were deleted to achieve the impact item below 1.5. During the qualitative content analysis, 5 items were deleted considering the experts’ judgment. For quantitative content validity, 7 items were deleted to obtain the CVR score below 0.49. Then, CVI was determined, where 4 expressions were deleted to obtain a score below 0.79. Accordingly, the number of expressions in the questionnaire was reduced to 45. Next, based on the mean CVI scores of all the expressions in the questionnaire, the mean CVI score of the questionnaire was calculated. It is worth mentioning that the mean CVI score of the questionnaire was as 0.91. Finally, the expressions were categorized into three groups including: human resources of clinical learning (29 expressions) learning conditions (4 expressions), and clinical learning strategies (12 expressions). 

After performing the required changes, to determine the construct validity of the items emerging from the interviews, we applied EFA on 45 expressions using the KMO and Cruet-Bartlett’s tests, where the KMO value of 0.856 was obtained. In addition, Cruet-Bartlett’s test with a value of 5937.891 was significant in the level of p=0.001 which justifies factor analysis implement based on covariance matrix on the studied samples (Table 2).

**Table 2:** Factor analysis, KMO and Bartlett’s test

** Test**	**Results**
Kaiser-Meyer-Olkin measure of sampling adequacy	0.856
Bartlett's test of sphericity Approx. Chi-Square	5937.891
df	990
Sig.	0.001

As mentioned in the findings, to determine the number of component factors in the questionnaire, we considered the cutoff point of 0.35 as the required loading for keeping the expression in the extracted factors, while the Eigen value of 2 was selected for determining the factors. The results indicated that the biggest share of the overall variance (40.55%) was defined by the 3 first factors while the rest of the variances (59.45%) was for the remaining 42 items. In other words, factor analysis indicated 3 factors with Eigen value above 2 which defines 40.55% of the variance. Therefore, 19.33%, 14.24%, and 6.98% of the covariance is defined by the first, second, and third factors, respectively (Table 3).

**Table 3:** Factor analysis, total variance estimated for three factors of effective factors of clinical learning instrument

**Component **	**Initial eigen values**			**Extraction sums of squared loadings**			**Rotation sums of squared loadings**		
	**Total**	**% of variance**	**Cumulative %**	**Total**	**% of variance**	**Cumulative %**	**Total**	**% of variance**	**Cumulative %**
1	12.418	27.596	27.596	12.418	27.596	27.596	8.697	19.327	19.327
2	3.321	7.381	34.977	3.321	7.381	34.977	6.405	14.234	33.561
3	2.508	5.572	40.550	2.508	5.572	40.550	3.145	6.989	40.550

Based on the rotated matrix of the components, factor 1 contains 5 components with negative loading, whereas factors 2 and 3 had the loading above 0.35, so they were placed in factors 2 and 3. Then, the 4 components with loading below 0.35 in all 3 factors were deleted based on the group decision, while the components with higher load values in each of these factors were placed in the corresponding category. Finally, 41 expressions were kept in the 3 factors, where factor 1 (instructor-staff related factors), factor 2 (student-related factors), and factor 3 (educational factors) contained 15, 13, and 13 statements, respectively (tables 4, 5 and 6). Reliability of this questionnaire calculated based on α-Cronbach coefficient was 0.93.

**Table 4:** Factor analysis, rotated matrix of the items for factor 1 (factors related to instructor-staffs)

**No.**	** Item**	**Loading **
1	Seriousness of the instructor has been effective on my learning	0.39
2	Calmness and pleasant behavior of the instructor has been effective on my clinical learning	0.79
3	Discipline of the instructor has been effective on my clinical learning	0.72
4	Indiscrimination of the instructor towards the students has been effective on my clinical learning	0.65
5	High self-confidence of the instructor has been effective on my clinical learning.	0.70
6	Specialty of the instructor in his teaching materials during the internship is effective on clinical learning of the student during	0.77
7	The knowledge and skill of the instructor significantly affect my clinical learning.	0.67
8	The efficient advice of the instructor has been effective on my clinical learning	0.69
9	Suitable feedback of my failures by the instructor has been effective on my clinical learning	0.47
10	Lack of unity among the instructors makes the students confused	0.37
11	Advice and guidance of the unit’s staff has been effective on my clinical learning	0.52
12	Appropriate and respectful behavior of the staff with me has been effective on my clinical learning	0.62
13	Out-of-date knowledge of the staff leads to inappropriate learning of the students.	0.94
14	Routine-based job performance by the staff prevents appropriate learning of the students	0.36
15	Confidence of the instructors and staff enhances my motivation towards clinical learning	0.45

**Table 5:** Factor analysis, rotated matrix of the items for factor 2 (factors related to students)

No.	** Item**	**Loading **
1	I try to learn the clinical jobs with more patience.	0.42
2	I try to reinforce my clinical weaknesses through the further curiosity.	0.41
3	I am in good physical conditions for the clinical jobs.	0.56
4	My interest in nursing results in my better clinical learning.	0.54
5	My responsibility towards the patient results in my better clinical learning.	0.51
6	My high self-confidence facilitates my better clinical learning.	0.55
7	My high pscycho-mental readiness results in my better clinical learning.	0.64
8	My adequate scientific knowledge results in my better clinical learning.	0.51
9	My religious values and beliefs result in my better clinical learning.	0.37
10	Interaction and communication among the students in the clinic leads to the better learning.	0.48
11	Applying the clinical experiences of other classmates leads to the better learning.	0.49
12	Performing the jobs independently and without supervision and support of the others enhances my learning ability.	0.56
13	Precise exploration of the patients’ problems and the factors related to their illness improves clinical learning.	0.53

**Table 6:** Factor analysis, rotated matrix of the items for factor 3 (factors related to education)

**No.**	**Item**	**Load factor**
1	Offering clinical learning course plan by the professors and clarifying the objectives of the internship course has been effective on my clinical learning.	0.44
2	Presence of a high number of students in the unit ruins the chance of clinical learning.	0.93
3	Offering the theoretical courses before the internship course facilitates the clinical learning.	0.52
4	Presence of multiple cases in the unit promotes my motivation toward further learning.	0.54
5	Performing the care giving procedures in clinic with patients promotes my clinical learning.	0.54
6	Selecting the patient by nurses’ own will has been effective on the clinical learning.	0.52
7	Scheduling and prioritizing the clinical care by the student leads to better learning.	0.93
8	Giving the library study time to the students in the unit can facilitate the clinical learning.	0.55
9	Offering the learning materials as group discussion enhances my clinical learning.	0.56
10	Presenting the scientific lectures in the unit enhances the clinical learning.	0.46
11	Offering the clinical report or presence of the instructors enhances the clinical learning.	0.50
12	Preparing the pamphlets and posters on the illness and the associated drugs significantly affects my clinical learning.	0.49
13	Holding learning programs through mobile enhances the clinical learning.	0.47

## Discussion

For a long time, many researches have been conducted on construction of the instruments used for clinical learning of the nursing students. In 1990’s, the audit tool was invented in England which had a strong emphasis on various aspects of clinical learning environment (16-18). Due to the fact that there is no common structure for the clinical learning, designing an instrument applicable in a wide range of countries seems to be a difficult task. This implies the importance of constructing an instrument which determines the students’ perspective on effective factors on clinical learning, in the cultural context of Iran. Marriott states that there are cultural differences in clinical learning organization in different countries (19).

In the qualitative part of this research, a questionnaire was designed for assessment of the effective factors on clinical learning based on clinical learning concept. This work is considered as an innovation because of designing and determining the psychometry of the instrument designed for assessment of the factors effective on clinical learning. The questionnaire can be conveniently used since it can be completed in 30 min by the students and instructors. 

First factor was the factors related to instructor-staff. Factors such as personal characteristics of the instructors (e.g seriousness, serenity, justice, self-confidence, and high level of knowledge), proper behavior of the staff with students and their updated knowledge are considered as the factors effective on students’ learning. The results reported by Chapman and Orb (2001) revealed that some characteristics of the instructors such as being supportive, encouraging, reference, reliable, available, friendly, useful, understanding, welcoming, and having inner interest in students are among the factors mentioned by students as promoter of their clinical learning level (20). Detecting the positive and negative characteristics of the instructors can assist clinical learning planners to select people with characteristics suitable for clinical learning of the students. Besides, in the case of observing particular negative characteristics they can see the feedback and avoid repeating them, or rather, they can be encouraged to present a positive personality. Moreover, about the effect of the staff on nursing students’ clinical learning , other works indicate that learning from the society and staff (21) which can be realized through the collaboration between nursing services and nursing education is another important factor (22). Kneafsey also believes that in order to acquire necessary clinical skills by the students, the nurses to treat them properly, spend time for them and talk to them, and teach them the required skills which enable them to make appropriate decisions in the clinical situations and appropriately deliver the healthcare services (23).

The second factor was related to the students. The findings of the present study indicated that curiosity, patience, responsibility, mental and physical readiness, having high knowledge, and interaction with classmates had highly affected the clinical learning of the students. Recognizing the above-mentioned factors is very important for the clinical instructors since students have their unique characteristics which should be pointed out by the instructor during the clinical clerkship. 

Interaction with classmates also plays a significant role in the learning process. White believes that factors such as learning from classmates are important in clinical learning (21). Paying attention to the social and organizational aspects of learning is more important than the individual learning since the horizontal learning is achieved through the interaction with colleagues (24). The third factor extracted from these data was educational factors. Here, precedence of the theoretical courses before the clerkship, clear duties of the students, number of student in the units, schedule and prioritization of the healthcare offered by the students, and presenting the clinical seminars are among the factors affecting the clinical learning process of the students. In a work conducted by Choy et al., the insufficient support offered by nursing department towards the educational field programs, inefficient supervision of the department and non-educational exploitation of the hospitals from students, not informing the educational authorities of the hospital about the new changes in the trend of clinical internship process, and not informing the hospital authorities about the interns’ duties were among the items mentioned by the authorities in the units (25). About precedence of theory to practice (internship), it must be noticed that the theoretical and academic learning enhance the students’ capabilities and prepare them for decision making in the clinical situations.

About presenting the clinical seminars and its effect on clinical learning, the clinical instructors can apply various strategies (such as desk studies, scientific conferences, group discussions, preparing the pamphlets, appropriate planning, etc.) to guide the students towards learning the desired theoretical knowledge. In another study, Stalmeijer et al. also constructed an instrument for evaluation of the clinical instructors, where factors such as practice and exercise, instructor’s support, respect to the student, and analysis of the clinical cases by the students were similar to those found in the present study (26).

## Conclusion

In conclusion, the results indicated that the constructed questionnaire is an efficient instrument for study of the effective factors on clinical learning in the view of nursing students since it involves 41 expressions and properties such as instrument design based on perceptions and experiences of the nursing students about the effective factors on clinical learning, definition of facilitating and inhibiting factors of the clinical learning, simple scoring, suitable validity and reliability, and applicability in different occasions. 

## References

[1] Daley BJ (2001). Learning in clinical nursing practice. Holistic Nursing Practice.

[2] Ogier ME (1989). Working and learning, the learning environment in clinical nursing.

[3] Spencer J (2003). Learning and teaching in the clinical environment. BMJ.

[4] Dunn SV, Hansford B (1997). Undergraduate nursing students' perceptions of their clinical learning environment. Journal of advanced nursing.

[5] Perli S, Brugnolli A (2009). Italian nursing students' perception of their clinical learning environment as measured with the CLEI tool. Nurse Education Today.

[6] Peyrovi H, Yadavar-Nikravesh M, Oskouie SF, Berterö C (2005). Iranian student nurses' experiences of clinical placement. Int Nurs Rev.

[7] Löfmark A, Wikblad K (2001). Facilitating and obstructing factors for development of learning in clinical practice: a student perspective. Journal of advanced nursing.

[8] Elliott M (2002). Clinical education: a challenging component of undergraduate nursing education. Contemporary Nurse.

[9] Hesse-Biber SN (2010). Mixed methods research, Merging theory with practice.

[10] Lacasse Y, Godbout C, Series F (2002). Health-related quality of life in obstructive sleep apnoea. European Respiratory Journal.

[11] Juniper EF, Guyatt GH, Streiner DL, King DR (1997). Clinical impact versus factor analysis for quality of life questionnaire construction. Journal of clinical epidemiology.

[12] Hyrkäs K, Appelqvist-Schmidlechner K, Oksa L (2003). Validating an instrument for clinical supervision using an expert panel. International journal of nursing studies.

[13] Polit DF, Beck CT, Owen SV (2007). Is the CVI an acceptable indicator of content validity? Appraisal and recommendations. Research in nursing & health.

[14] Munro BH (2005). Statistical methods for health care research.

[15] Knapp TR, Brown JK (1995). Ten measurement commandments that often should be broken. Research in Nursing & Health.

[16] Shailer B (1990). Clinical learning environment audit. Nurse Education Today.

[17] Reed S, Price J (1991). Nurse Education: Audit of clinical learning areas. Nursing times.

[18] Farrell GA, Coombes L (1994). Student nurse appraisal of placement (SNAP): an attempt to provide objective measures of the learning environment based on qualitative and quantitative evaluations. Nurse Education Today.

[19] Marriott A (1991). The support, supervision and instruction of nurse learners in clinical areas: a literature review. Nurse Education Today.

[20] Chapman R, Orb A (2001). Coping strategies in clinical practice: The nursing students' lived experience. Contemporary Nurse.

[21] White C (2010). A socio-cultural approach to learning in the practice setting. Nurse Education Today.

[22] Rich KL, Nugent KE (2010). A United States perspective on the challenges in nursing education. Nurse Education Today.

[23] Kneafsey R, Haigh C (2007). Learning safe patient handling skills: Student nurse experiences of university and practice based education. Nurse Education Today.

[24] Engestrom Y (2001). Expansive learning at work: Toward an activity-theoretical reconceptualization. Journal of education and work.

[25] Choy S, Delahaye B (2011). Partnerships between universities and workplaces: some challenges for work-integrated learning. Studies in Continuing Education.

[26] Stalmeijer RE, Dolmans DH, Wolfhagen IH, Muijtjens AM, Scherpbier AJ (2008). The development of an instrument for evaluating clinical teachers: involving stakeholders to determine content validity. Med Teach.

